# Parasitization of the pedicle: a rare form of TRAM flap recurrence

**DOI:** 10.1093/jscr/rjad322

**Published:** 2023-07-31

**Authors:** Matthew J Binks, CiCi Guo, Danusha Sabanathan, Sivapirabu Sathasivam, Senarath Edirimanne

**Affiliations:** Department of Surgery, Gosford Hospital, Gosford, NSW, Australia; University of Newcastle, Newcastle, NSW, Australia; Department of Surgery, Nepean Hospital, Kingswood, NSW, Australia; Department of Surgery, Nepean Hospital, Kingswood, NSW, Australia; Department of Surgery, Nepean Hospital, Kingswood, NSW, Australia; Department of Surgery, Nepean Hospital, Kingswood, NSW, Australia; University of Sydney, Sydney, NSW, Australia

**Keywords:** breast cancer, local recurrence, breast reconstruction

## Abstract

Local recurrence after mastectomy and autologous breast reconstruction is uncommon and tends to occur predictably within the superficial tissues or at the chest wall. We present a unique case of breast cancer recurrence involving the superficial and deep tissues. By parasitizing the pedicle of a free transverse rectus abdominis myocutaneous flap pedicle, the tumour was seen to extend through the chest wall to the right pleura.

## INTRODUCTION

Local recurrence of breast cancer after mastectomy and breast reconstruction is uncommon. If local recurrence occurs, it is typically within the superficial skin envelope or, infrequently, at the chest wall [1, 2]. We present a rare case of recurrence involving parasitization of a transverse rectus abdominis myocutaneous (TRAM) flap pedicle, whereby a cancer has spread from the skin envelope, through the chest wall to the pleura along the flap’s vascular pedicle.

## CASE REPORT

In October 2014, a 39-year-old woman, RC, presented with a 2-month history of right breast pain and an associated mass. RC had a strong family history of breast cancer. The core biopsy confirmed high grade ductal carcinoma in situ (DCIS). Bilateral nipple sparing mastectomies (NSM) with single-stage immediate implant reconstruction were performed in November 2014. Histopathology diagnosed a 100 mm high grade DCIS, which was focally present at the superficial margin. The patient received adjuvant radiotherapy to the reconstructed right breast.

In November 2015, RC developed a right-sided symptomatic capsular contraction. Two months later, this was managed with a capsulotomy and implant exchange. Due to ongoing capsular contracture in December 2018, her reconstruction was revised to bilateral free TRAM flaps, which were anastomosed to the internal mammary vessels bilaterally.

**Figure 1:**
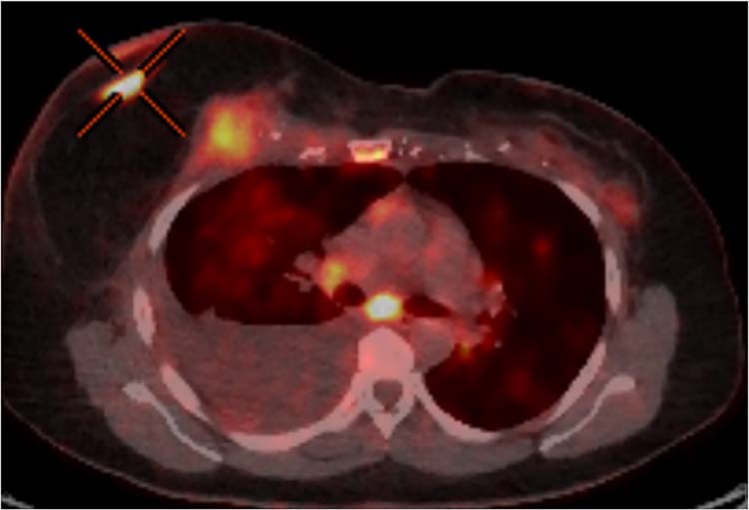
PET-CT axial slice showing both superficial and deep components of the local recurrence with associated malignant pleural effusion from direct extension to the pleural cavity.

In June 2020, RC noted changes to the skin of her right breast. These changes were associated with a palpable mass deep to her nipple areolar complex. Biopsy of this lesion found it to be recurrent invasive ductal carcinoma, which was hormone receptor negative and HER2 receptor positive. MRI and PET imaging traced the lesion from the skin flap, via the TRAM flap pedicle, into the thoracic cage to involve the pleura (Fig. 1). Regional metastases to the right axillary lymph nodes and distant metastases to the mediastinal nodes, lungs bilaterally and spine were evident.

Multidisciplinary input was sought and taxol chemotherapy in combination with trastuzumab and pertuzumab anti-HER2 therapy was implemented. In January 2021, a complete clinical and radiological response was achieved.

## DISCUSSION

Over the past three decades, the deep inferior epigastric perforator (DIEP) free flap and the TRAM free flap have been instrumental in breast reconstruction. Both procedures involve harvesting an ellipse of subcutaneous fat and skin from the lower abdomen, with or without the underlying rectus abdominis muscle—TRAM and DIEP, respectively—based on the inferior epigastric vessels. These vessels are commonly anastomosed to the internal mammary vessels after resection of the second or third costal cartilage at the sternal edge of the affected side.

To our knowledge, this case study details a pattern of recurrence and local invasion unique in the literature. The exact pattern of spread is unclear and highlights the uncertain mechanisms of local recurrence following breast reconstruction. First, it is likely that the initial recurrence was superficial with subsequent extension to deeper tissues. The mechanism of spread from a superficial recurrence to the deeper tissues of a breast reconstruction has been examined previously [3]. Possible explanations include direct invasion of the deeper tissues or transit of metastatic cells through new lymphatic channels created by the reconstructive surgery [4]. Furthermore, the flap’s vascular pedicle, in conjunction with the required rib resection to facilitate its anastomosis to the internal thoracic vessels, has likely provided a conduit for tumour spread between the superficial tissues of the chest wall and the deeper structures of the thoracic cage, in a process of parasitization of the vascular pedicle.

Local recurrence of breast cancer affects 1–7% of mastectomies and significantly impacts patient survival [1, 5, 6]. The retention of the skin envelope in skin (SSM) and NSM potentially increases the risk of local recurrence. Furthermore, SSM and NSM have an increased risk of incomplete mastectomy, with 13.2% and 51% of patients having retained breast tissue post-procedure, respectively, compared with 2.8% of people receiving simple mastectomy [7].

Local recurrence after breast reconstruction can occur superficially in the skin envelope, deeply at the chest wall or within the flap itself. Superficial recurrence accounts for 80–87.5% of recurrences, with deep disease making up the remainder [1, 2]. Superficial recurrence portends a favourable prognosis when compared with chest wall recurrences. Approximately 61% of patients with a superficial recurrence survive to 80 months, compared with 45% of those with chest wall recurrences [2].

We present a unique case of breast cancer recurrence involving the superficial and deep tissues, the tumour having spread to the pleural space by parasitizing a TRAM flap pedicle.

## Data Availability

The data that support the findings of this study are available on request from the corresponding author (MB). The data are not publicly available due to their containing information that could compromise the privacy of research participant.
